# Enhancing Flexural Behavior of Reinforced Concrete Beams Strengthened with Basalt Fiber-Reinforced Polymer Sheets Using Carbon Nanotube-Modified Epoxy

**DOI:** 10.3390/ma17133250

**Published:** 2024-07-02

**Authors:** Changchun Shi, Shengji Jin, Chengjie Wang, Yuhao Yang

**Affiliations:** 1 School of Materials Science and Engineering, Shenyang University of Technology, Shenyang 110870, China; 2 Department of Civil Engineering, Hebei University of Water Resources and Electric Engineering, Cangzhou 061001, China; 3 School of Architecture and Civil Engineering, Shenyang University of Technology, Shenyang 110870, China

**Keywords:** premature debonding failure, reinforced concrete beams, basalt fiber-reinforced polymer, flexural behavior, multi-wall carbon nanotubes, epoxy resin

## Abstract

The external bonding (EB) of fiber-reinforced polymer (FRP) is a usual flexural reinforcement method. When using the technique, premature debonding failure still remains a factor of concern. The effect of incorporating multi-wall carbon nanotubes (MWCNTs) in epoxy resin on the flexural behavior of reinforced concrete (RC) beams strengthened with basalt fiber-reinforced polymer (BFRP) sheets was investigated through four-point bending beam tests. Experimental results indicated that the flexural behavior was significantly improved by the MWCNT-modified epoxy. The BFRP sheets bonded by the MWCNT-modified epoxy more effectively mitigated the debonding failure of BFRP sheets and constrained crack development as well as enhanced the ductility and flexural stiffness of strengthened beams. When the beam was reinforced with two-layer BFRP sheets, the yielding load, ultimate load, ultimate deflection, post-yielded flexural stiffness, energy absorption capacity and deflection ductility of beams strengthened using MWCNT-modified epoxy increased by 7.4%, 8.3%, 18.2%, 22.6%, 29.1% and 14.3%, respectively, in comparison to the beam strengthened using pure epoxy. It could be seen in scanning electron microscopy (SEM) images that the MWCNTs could penetrate into concrete and their pull-out and crack bridging consumed more energy, which remarkably enhanced the flexural behavior of the strengthened beams. Finally, an analytical model was proposed for calculating characteristic loads and characteristic deflections of RC beams strengthened with FRP sheets, which indicated a reasonably good correlation with the experimental results.

## 1. Introduction

Fiber-reinforced polymer (FRP) has been widely used in the flexural strengthening of reinforced concrete (RC) beams due to its high tensile strength, low density and ease of installation [1,2,3,4]. The external bonding (EB) technique of FRP is a usual flexural reinforcement method. Premature debonding failure is the major disadvantage of the EB technique, which reduces the strengthening efficacy [5,6,7,8].

Many efforts have been made to mitigate premature debonding failure of FRP sheets. Cheng Yuan et al. [9] investigated epoxy anchors to mitigate the debonding process of basalt fiber-reinforced polymer (BFRP) sheets of strengthened RC beams, in which the displacement and ultimate load increased by 53.31% and 13.12%, respectively. Ayman N. Ababneh et al. [10] reported that the FRP-anchored beams with the bolted steel plates experienced concrete cover separation failure, while the unanchored beams experienced premature debonding. A grooving technique was utilized to enhance the flexural capacity and ductility of RC beams reinforced using FRP sheets [11]. However, in some cases, in order to protect fragile concrete [12] or to prevent damage to the rebar [13,14], anchorage measures or the grooving technique might not be available and the EB technique must be applied. Consequently, it is necessary to improve the epoxy resins commonly used as adhesives in order to mitigate the premature debonding.

Various kinds of nanofillers can be incorporated in epoxy resins for enhancing their mechanical performance [15,16,17]. As a kind of nanomaterials, carbon nanotubes (CNTs) have excellent mechanical and physical properties due to their geometric arrangement and their strong sp^2^ carbon–carbon bondings and have attracted extensive attention [17,18,19]. For example, epoxy resins modified by CNTs have been used to improve the mechanical properties of many types of FRP sheets [20,21,22,23,24]. Many studies have shown that the adhesive properties of adhesive joints can be improved by incorporating various nanofillers into epoxy resins [12,25,26,27]. Our previous study [12] reported that adding multi-wall carbon nanotubes (MWCNTs) to epoxy significantly mitigated debonding failure of BFRP sheets in the single-shear tests.

However, the stress state of FRP sheets is different in single shear tests and beam surfaces, and few studies have investigated the effect of CNTs added to epoxy resin on the flexural behavior of RC beams strengthened with FRP sheets. Irshidat M. R. et al. [28] experimentally studied the role of MWCNTs in enhancing the flexural properties of RC beams reinforced with CFRP sheets, which showed that the ultimate load as well as the stiffness of retrofitted beams were enhanced using CNT-modified epoxy resin. However, the strain response of CFRP, steel rebar, concrete, as well as the development process of crack width were not investigated in this study.

With this background in mind, the present study aimed to investigate the effect of CNT-modified epoxy on the flexural behavior of RC beams strengthened with externally bonded FRP sheets. The proposed research results may be applied in the rehabilitation and strengthening of RC structures using externally bonded FRP sheets. In this study, the flexural behavior of RC beams strengthened with BFRP sheets using MWCNT-modified epoxy or neat epoxy was studied through four-point bending beam tests, including failure mode, load–deflection relationship, development process of crack width, energy absorption capacity, stiffness, ductility as well as the strain response of the BFRP sheet, rebar and concrete. The experimental variables included the quantity of BFRP layers and type of adhesive. The enhancement mechanisms of the MWCNTs on the flexural behavior of RC beams strengthened with BFRP sheets were analyzed by scanning electron microscopy (SEM) observations. Finally, an analytical model was proposed for calculating characteristic loads and characteristic deflections of RC beams strengthened with FRP sheets.

## 2. Experimental Program

### 2.1. Material Properties

The concrete strength grade was specified as C30 and its cubic compressive strength, axial compressive strength, axial tensile strength and compressive elastic modulus were 37.8 MPa, 26.1 MPa, 2.66 MPa and 31.1 GPa, respectively. Table 1 shows the mechanical properties of the rebar used for the tested beams.

**Figure 1:**
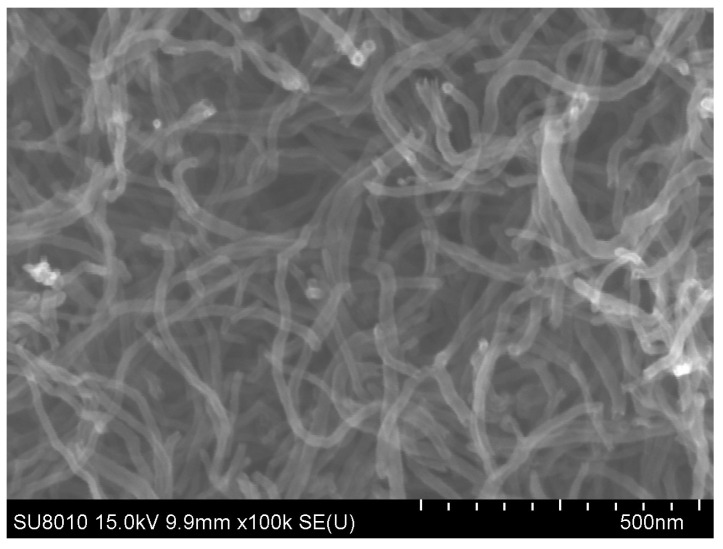
Morphology of MWCNTs.

**Figure 2:**
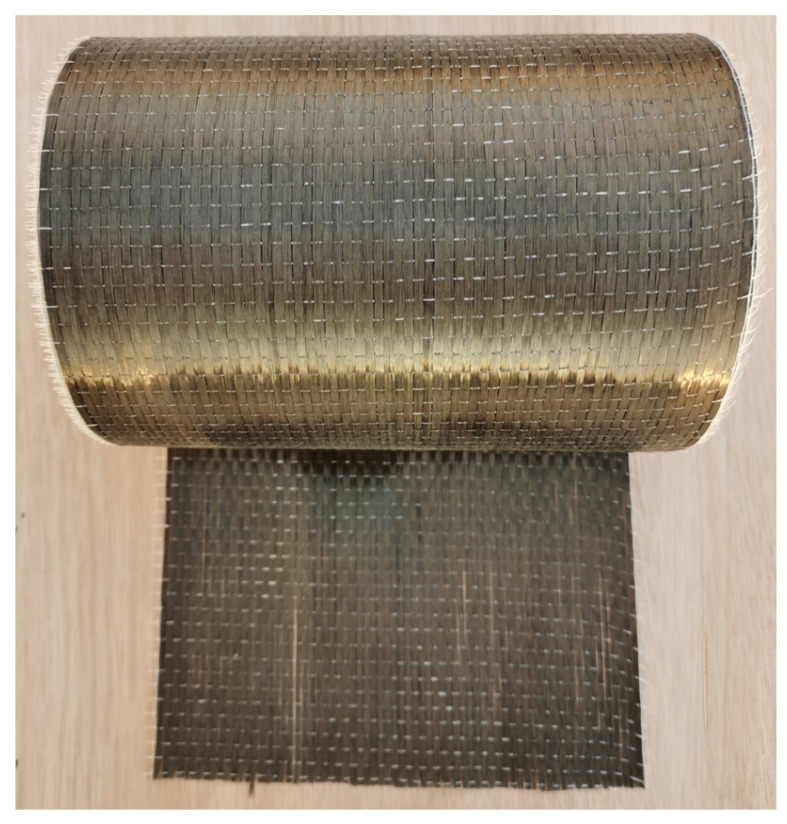
The unidirectional basalt fiber fabric.

BFRP sheets were selected for this study because of their superior mechanical performance, good corrosion resistance, and environmental friendliness [29,30,31,32]. The Industrial-grade COOH-MWCNTs (external diameter of 20–30nm and length of about 10–30 μm) were selected in order to disperse the MWCNTs evenly in the epoxy [33]. An SEM image of the COOH-MWCNTs was shown in Figure 1. The area density and thickness of basalt fiber fabric (Figure 2) were 300 g/m^2^ and 0.12 mm, respectively. The tensile strength, elastic modulus and ultimate strain of the BFRP without MWCNTs were 1313 MPa, 71 GPa and 1.86%, respectively. However, the tensile strength, elastic modulus and ultimate strain of the BFRP with MWCNTs were 1339 MPa, 77 GPa and 1.74%, respectively. The mass ratio of the curing agent and bisphenol-A epoxy was 1:1. The ultimate tensile strength, ultimate rupture strain and elastic modulus of the epoxy resin were 50.5 MPa, 4.5% and 2.8 GPa, respectively.

### 2.2. Preparation of Test Specimens

**Figure 3:**
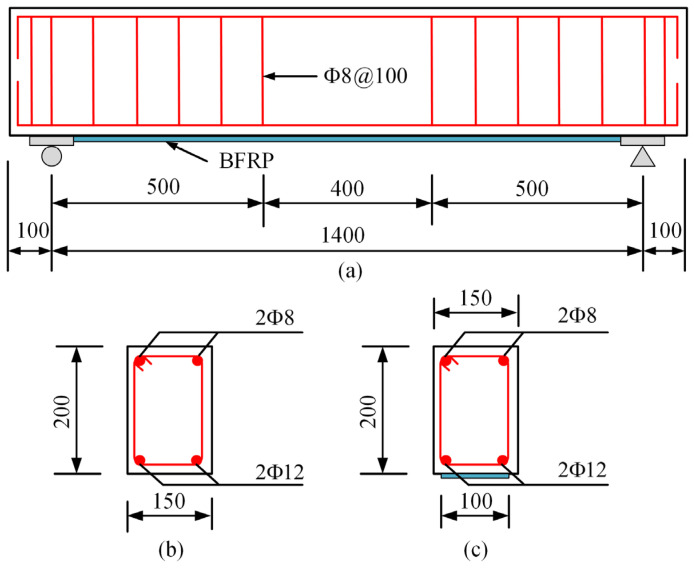
Dimensions and reinforcement details of the test specimens: (**a**) beam geometry; (**b**) reference beam cross-section; and (**c**) strengthened beam cross-section (all dimensions are in mm).

Five RC beams were prepared, and the dimension of each RC beam specimen was 150 × 200 × 1600 mm and clear span was 1400 mm. The RC beams were designed according to code GB50010-2010 [34]. Due to limitations of laboratory equipment, it was not possible to fabricate full-scale beams, and hence these beams were designed as scaled down specimens of typical beam elements in practice [9,10,35]. The details of the specimen are presented in Figure 3. All beams with a concrete cover thickness of 25 mm were constructed using the same strength grade and quantity of concrete and rebar. Two steel bars with a diameter of 8 mm and two steel bars with a diameter of 12 mm were used in the longitudinal direction of the top and bottom, respectively. The beam samples were reinforced by steel stirrup with a diameter of 8 mm and a central spacing of 100 mm in the shear span region.

All the five tested beams consisted of one reference unreinforced RC beam and four RC beams strengthened using BFRP sheets. The four RC beams were strengthened with BFRP sheets according to two design codes, GB50367-2013 and GB50608-2010 [36,37]. The BFRP sheets with a width of 100 mm and a length of 1400 mm were attached to the bottom surface of the beams. Table 2 lists the designation and description of every beam specimen. RC beam CB was employed as a reference specimen.

**Figure 4:**
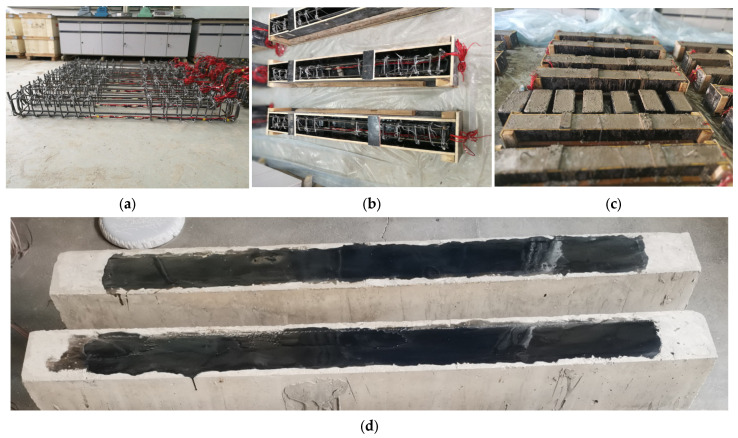
Manufacturing process of the test beam: (**a**) the inner reinforcement cage; (**b**) placing the tied cage in the formwork; (**c**) specimens after concrete cast; and (**d**) bonding the bottom tension BFRP sheets.

The neat epoxy resin had a 1:1 weight proportion of curing agent to resin. The MWCNT-modified epoxy contained 0.8 wt.% of MWCNTs dispersed in epoxy resin by magnetic stirring followed by an ultrasonic bath [12]. The manufacturing process of the test beams is exhibited in Figure 4. Firstly, the steel bars were processed according to the design dimensions of the test beam and fixed with hoop ties to form the reinforcement cage. The wooden formwork was then made according to the dimensions and the tied cage was placed in the wooden formwork. Once the preparation work had been completed, the concrete for the test beam was ready for casting. After the completion of the concrete pouring, the test beams were cured for 28 days. Before bonding the BFRP sheets to remove the superficial mortar layer, the concrete surfaces were rubbed by an angle grinder. The grit particles on the bonding surfaces were sucked by a vacuum cleaner. After being wiped with alcohol pads, the BFRP sheet was pasted to the beam surface by a wet lay-up procedure. The specimens were cured for more than two weeks under normal indoor conditions before loading.

### 2.3. Testing and Loading Solutions

**Figure 5:**
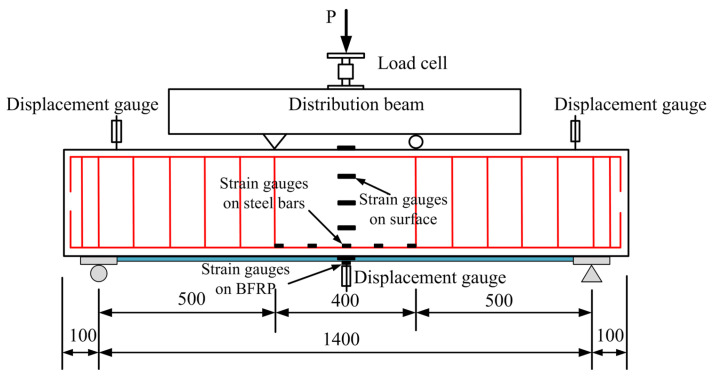
Schematic diagram of loading (all dimensions are in mm).

**Figure 6:**
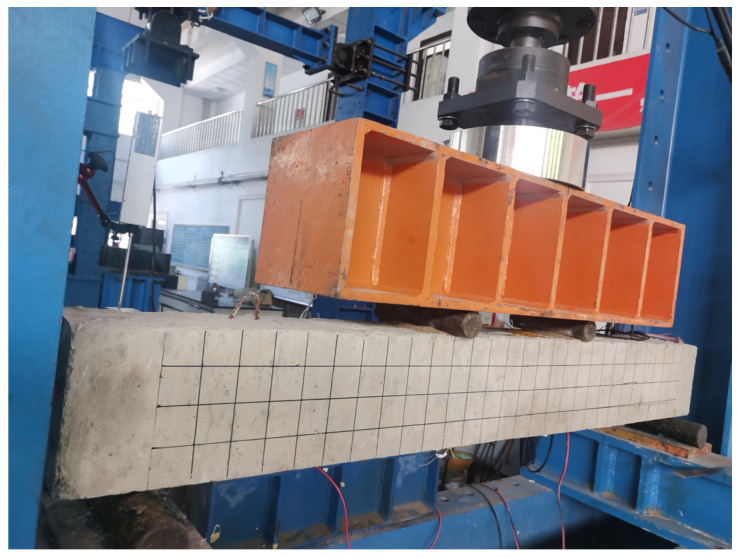
Four-point bending test setup.

All beams were simply supported and were tested under four-point bending with shear spans of 500 mm. Figure 5 and Figure 6 illustrate the schematic diagram of loading and test setup. To all beams the load was applied under a force control scheme using a hydraulic servo machine with a capacity of 500 KN through a steel distribution beam. The loading changes in real time were monitored using a 1000 KN load cell. Three YDH-100 electronic displacement gauges were placed to measure the mid-span deflection and supports of each beam. The steel strain in the pure bending section was monitored through strain gauges affixed on the surface of the longitudinal steel bars. The strain gauges were also attached to the concrete surface and BFRP surface at the beam’s mid-span. The data of strain, displacement and load were recorded and saved using the data acquisition system. In addition, the crack widths under different loads were measured by a crack-width meter.

Before the test beam was formally loaded, it was necessary to carry out pre-loading to test whether the loading device was in close contact with the load sensor, the distribution beam and the test beam. The pre-load value was 5% of the calculation values of the ultimate load. During pre-loading, it was necessary to observe the reading of the load sensor and the displacement meter on the data acquisition system to determine whether the data acquisition equipment worked normally. After the check was correct, the load was removed. If any problem was found during the pre-loading process, it was necessary to test and debug again after unloading until everything was normal. In the process of formal loading, the loading rate of this test was first controlled by force, and then by displacement. The loading value of each stage was controlled to 10% of the calculation values of the ultimate load at a control rate of 0.1 KN/s, and each load stage was maintained for 10 min, the purpose of which was to ensure that the crack, deflection and strain fully developed and tended to be stable, so as to facilitate the measurement of crack width and the drawing of crack development trend. When the loading value reached 85% of the calculation values of the ultimate load, the loading was continued at a rate of 0.5 mm/min under displacement control until the test beam failed.

## 3. Experimental Results and Discussions

### 3.1. Load–Deflection Response

**Figure 7:**
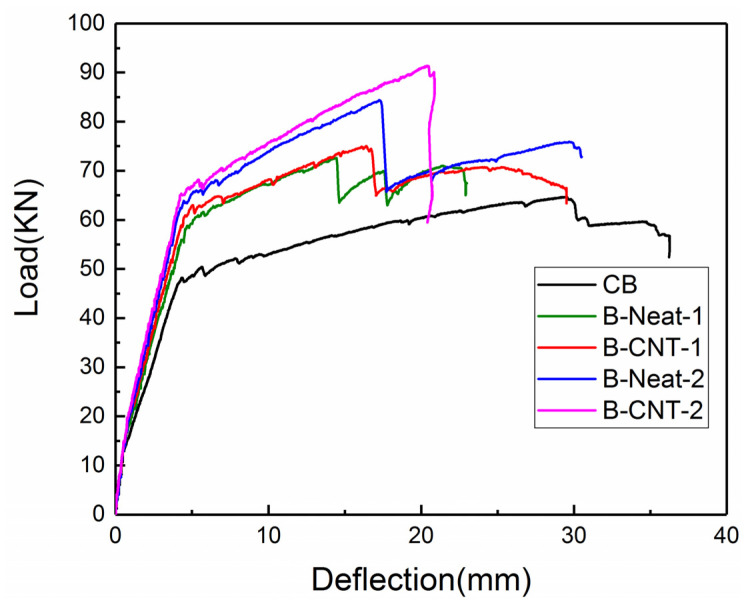
Load versus mid-span deflection curves of the tested beams.

Table 3 presents a summary of the characteristic deflections, characteristic loads and flexural stiffness of all beams. The load versus mid-span deflection curves of all the specimens are plotted in Figure 7, indicating that each curve was divided into three distinct stages: the elastic stage from the beginning of loading to concrete cracking, the elastic–plastic stage of initiation of cracks up to yielding, as well as the plastic stage after yield. All beams presented similar elastic behavior from loading to concrete cracking in the first stage, during which the beam reinforced using MWCNT-modified epoxy resin exhibited a slightly higher cracking load and did not show a significant enhancement. The strengthening effect of the MWCNT-modified epoxy resin was more obvious in the latter two stages, where the curve slope of the beams strengthened using MWCNT-modified epoxy became steeper in comparison to the beams strengthened using neat epoxy in the case of the same number of BFRP layers, which was in accordance with experimental results of a previous study [28].

Compared with the CB specimen, the ultimate loads of B-Neat-1, B-CNT-1, B-Neat-2 and B-CNT-2 increased by 12.2%, 15.9%, 30.3% and 41.4%, respectively; in addition, the yield loads of B-Neat-1, B-CNT-1, B-Neat-2 and B-CNT-2 increased by 29.1%, 36.5%, 45.3% and 50.5%, respectively. For the beams strengthened using same BFRP layers, the remarkable enhancement of the yielding load, ultimate load, ultimate mid-span deflection, as well as flexural stiffness *E_I_* and *E_II_* of the strengthened beams could be achieved by using the MWCNT-modified epoxy. For example, the yielding load, ultimate load, ultimate deflection as well as post-yielded flexural stiffness of the specimen B-CNT-2 increased by 7.8%, 8.5%, 8.7% and 22.6%, respectively, in comparison to the specimen B-Neat-2. When the beams were strengthened using the identical adhesive, increasing the number of BFRP layers could also provide similar enhancement. Compared with the sample B-CNT-1, the yielding load, ultimate load, ultimate deflection as well as post-yielded flexural stiffness of the sample B-CNT-2 increased by 10.3%, 22.0%, 24.5% and 29.3%, respectively.

### 3.2. Failure Mode and Cracking Development

**Figure 8:**
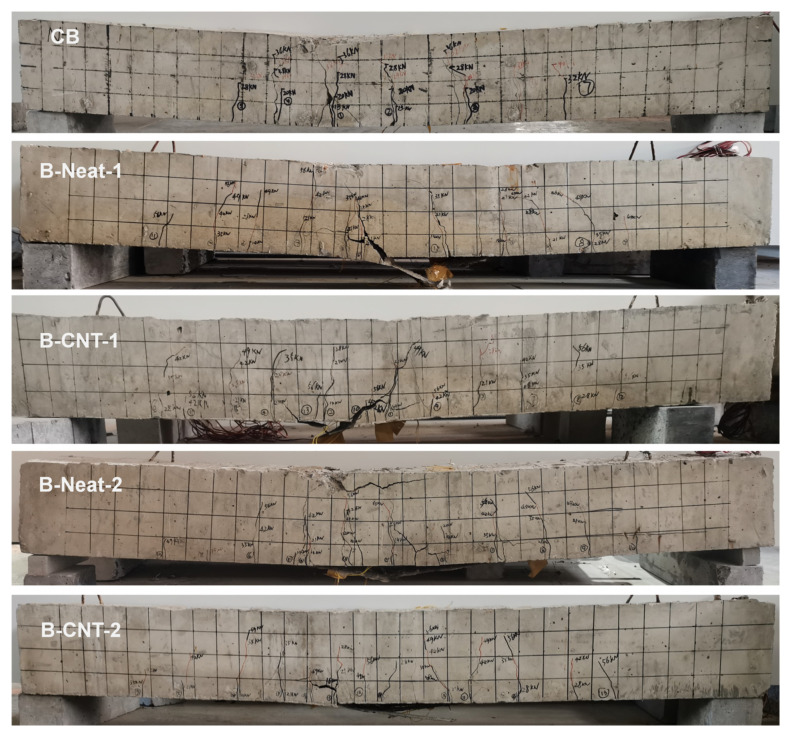
Failure modes and crack distribution.

**Figure 9:**
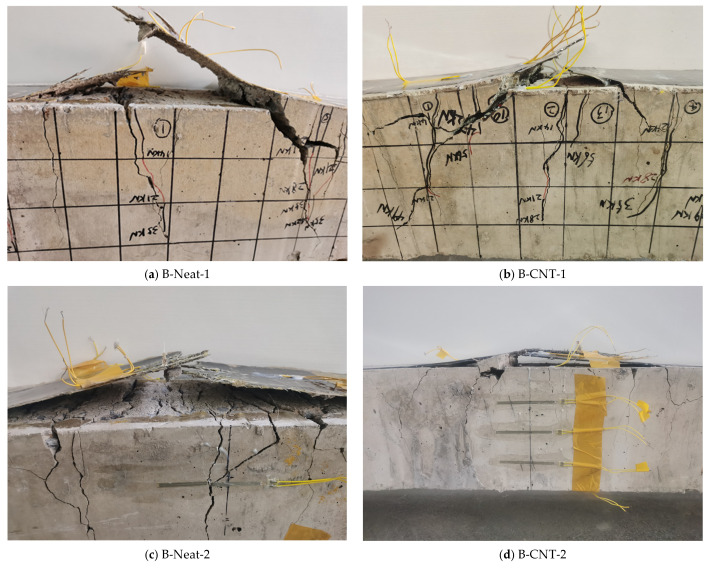
Zoomed view of failure zones in the strengthened beams.

The failure modes and crack distribution of the control and strengthened beams are shown in Figure 8. Figure 9 shows a zoomed view of the failure of the strengthened beams. CB beam failed in a representative ductile bending mode. The vertical cracks were triggered in the high moment region, followed by yield of the reinforcement as well as concrete crushing.

The reinforced specimens failed due to intermediate crack debonding (IC debonding) after steel yielding. IC debonding failure initiated at the toe of a major crack in the high moment region and spread towards the low moment region. A layer of concrete materials, a few millimeters thick, was attached to the debonded BFRP sheets, which indicated that the failure occurred within the concrete [38], as shown in Figure 9. Under the ultimate load, the maximum measured strain (i.e., debonding strain) of the BFRP sheets at mid-span was from about 4900 to approximately 9600 με, which was much lower than the rupture strain of the BFRP sheet. At this time, the concrete compressive strain was about 2000~3200 με, which did not reach the ultimate compressive strain. For the beams strengthened using same BFRP layers, the concrete strain of the strengthened specimens using MWCNT-modified epoxy was greater than that of the strengthened specimens using neat epoxy resin at the corresponding ultimate load. Subsequently, for the specimens B-Neat-2, B-Neat-1 and B-CNT-1, the BFRP sheets were ruptured and the load dropped to a low value, and then the load increased slowly until concrete crushing. For the specimen B-CNT-2, after the ultimate load, the load dropped slightly and then remained almost constant, and the concrete strain continued to increase until concrete crushing. The subsequent load continued to decrease until the final FRP fracture, at which point the load was 59.46 KN. The failure mode was also mentioned in previous studies [39,40]. Although the IC debonding was still premature debonding, the MWCNT-modified epoxy delayed the IC debonding as well as eventual fracture of BFRP, which increased the ultimate load and ductility.

**Figure 10:**
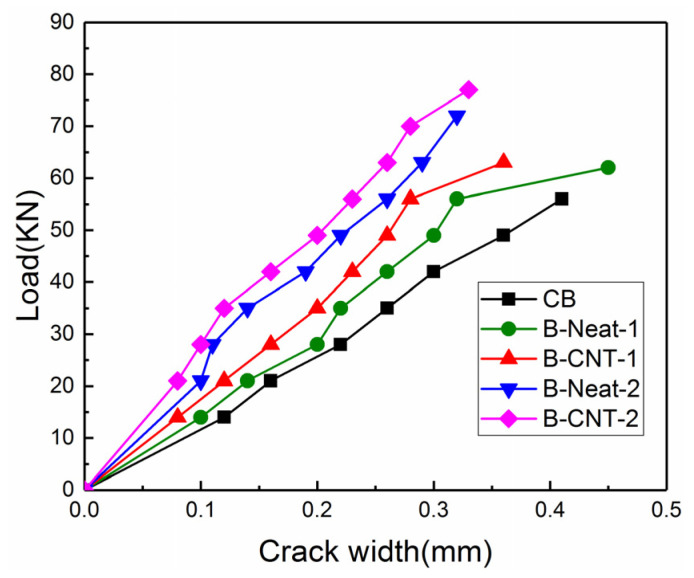
Load versus crack width.

There were more vertical flexural cracks with smaller width in the middle part of the strengthened beam, which indicated that the BFRP sheets controlled the crack widths. The same result was reported in the literature [41,42,43]. Not only the MWCNTs incorporated into epoxy resin but also the quantity of BFRP layers had remarkable effects on reducing crack width. The development of crack width prior to 85% of ultimate load is shown in Figure 10. Under the same load, for the beams strengthened with the same adhesive, the maximum crack width of the beams reinforced with two layers of BFRP sheets was smaller than that of the beams with single layer of BFRP sheet. In addition, when subjected to the same load, for the beams strengthened using the same BFRP layers, the maximum crack width of the beams using pure epoxy was greater than that of the beams using MWCNT-modified epoxy. The addition of MWCNTs constrained crack development and delayed the debonding of BFRP sheets, which might be attributed to the improvement of the concrete–epoxy interface because of the incorporation of MWCNTs [28,41,44].

### 3.3. Strain Analysis

**Figure 11:**
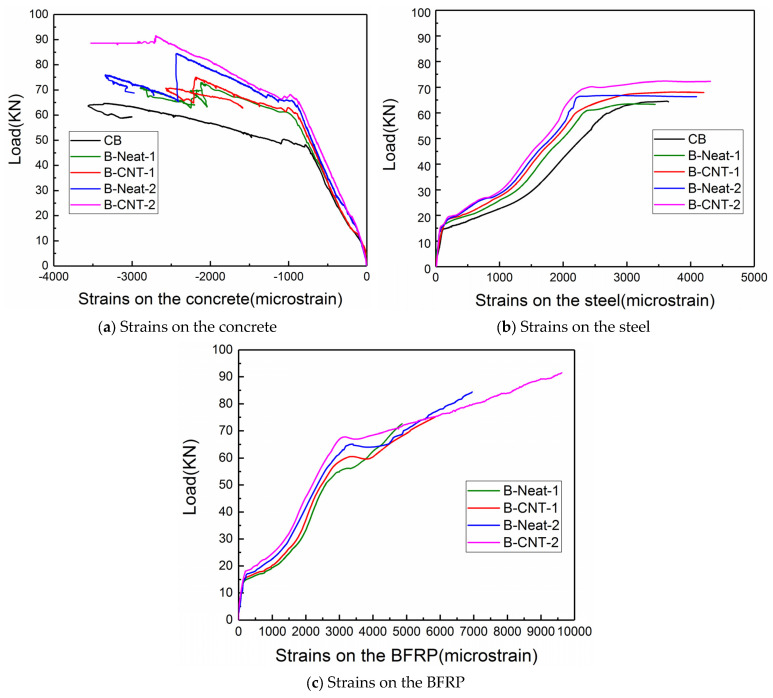
Load–strain curves of the beams.

Figure 11 exhibits the load–strain response for the top concrete, tensile longitudinal rebar and BFRP sheets at the beam’s mid-span. Both the MWCNTs and the quantity of BFRP layers had an obvious effect on the post-cracking strain behavior. Prior to cracking, the concrete of all the reinforced beams exhibited a similar strain response, as shown in Figure 11a. After the concrete cracking, at the same load level, the concrete strains of the reference beam CB were slightly greater than those of the reinforced beams. For the beams strengthened using the same BFRP layers at the same load, the concrete strains of the beams strengthened with neat epoxy were greater than those of the beams reinforced with MWCNT-modified epoxy. This was associated with the fact that the BFRP with MWCNT-modified epoxy increased the flexural stiffness, resulting in smaller deformation and strain. For the beams strengthened using same BFRP layers, the concrete strain of the specimens strengthened using MWCNT-modified epoxy was greater than that of the specimens strengthened with neat epoxy resin at the corresponding ultimate load.

In Figure 11b, the strain of the rebar exhibits a linear elastic behavior before the concrete cracking. When cracks occurred in concrete, the strain of the rebar increased suddenly. In view of the strengthening effect of BFRP sheets, under the same load, the strains of the rebar were lower than those of the unstrengthened beam. The strengthening effect was more significant for the BFRP sheets bonded using MWCNT-modified epoxy.

From Figure 11c, it can be seen that before concrete cracking, the strain in the BFRP sheets maintained a linear growth trend although it was minimal, and the slopes of the curves had little difference. After that, the strains of the BFRP sheet began to increase more significantly, demonstrating the contribution of BFRP to bearing load. Under the same load, the BFRP sheet strains of beams strengthened using two layers of BFRP sheets were slightly lower than those of of beams using one layer of the BFRP sheet. In the case of the same quantity of BFRP layers and at an identical load level, the BFRP strains of the reinforced beams with neat epoxy were greater than those of the beams reinforced with MWCNT-modified epoxy. For the beams strengthened using same BFRP layers, at the corresponding ultimate load, the debonding strain of BFRP sheets of the beams strengthened with pure epoxy was less than that of the beams strengthened with MWCNT-modified epoxy. The results showed that the use of MWCNT-modified epoxy delayed IC debonding, resulting in a significant increase in FRP utilization rate.

### 3.4. Energy Absorption Analysis

By integrating the area under the load–deflection curve, the energy absorption capacity (J) is obtained [45,46,47,48]. Table 4 lists the J value before a certain characteristic load of all beams. The J value was greatly affected by the type of adhesive. The J value before the ultimate load of B-CNT-1 has a 19.9% increase in comparison to B-Neat-1. Compared with the specimen B-Neat-2, the energy absorption before the ultimate load the specimen B-CNT-2 increased by 29.1%. For the beams strengthened using same BFRP layers, the beams strengthened using neat epoxy had lower J values than the beams strengthened with MWCNT-modified epoxy at the corresponding load.

### 3.5. Ductility Analysis

An RC beam with high ductility can undergo greater plastic deformation without significant strength reduction [49,50]. Ductility is an important parameter and can be evaluated according to deformation or energy [41]. Two ductility indexes ($\mu_{E}$ and $\mu_{\Delta }$) are calculated in terms of Equations (1) and (2) [41,49].

Energy ductility index:

(1) $${\;\;\mu }_{E}=\frac{J_{u}}{J_{y}}$$

Deflection ductility index:

(2) $$\mu_{\Delta }=\frac{{∆}_{u}}{{∆}_{y}}$$

The ductility indexes are computed and showed in Table 5. These data show that the unreinforced beam was more ductile than all the reinforced beams. For the beams strengthened using same BFRP layers, the ductility of the beams strengthened with neat epoxy was significantly lower than that of the reinforced specimens with MWCNT-modified epoxy. When using the same adhesive, the beams strengthened using two layers of BFRP were more ductile than those with a single layer of the BFRP sheet.

### 3.6. Analysis of Enhancement Mechanism of MWCNTs Modified Epoxy

**Figure 12:**
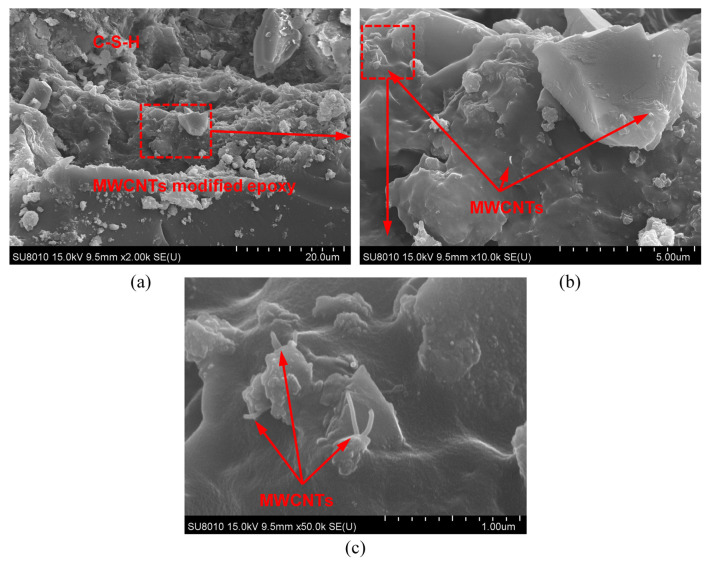
SEM images of fracture surface of specimen B-CNT-2: (**a**) 2000×, (**b**) 10,000× and (**c**) 50,000×.

To explore the role of MWCNTs in mitigating debonding failure of RC beams bonded with BFRP sheets, SEM observation was carried out at the debonding sheet of the specimen B-CNT-2. The MWCNTs could be found in the concrete pores by SEM images at the fracture surfaces of the specimen B-CNT-2 in Figure 12, which indicated the MWCNTs could permeate into the concrete pores, and the calcium–silicate–hydrate (C–S–H) was combined with MWCNT-modified epoxy. Both pull-out and crack bridging of the MWCNTs increased the interfacial fracture energy and delayed debonding initiation and propagation of BFRP sheets. Consequently, the MWCNT-modified epoxy remarkably mitigated debonding failure and enhanced bending performance of the strengthened beams.

## 4. Analytical Predictions

Based on the ACI Code, an analytical study was conducted to evaluate the accuracy of the equations to calculate characteristic loads and characteristic deflections of the RC beams strengthened with FRP sheets using MWCNT-modified epoxy.

### 4.1. Calculation of Flexural Capacity in Characteristic Points

The representative load versus deflection curve of an RC beam can be schematically divided into three distinct stages [9]: (I) Pre-cracking stage $\left(P<P_{cr}\right);$ (II) initiation of cracking up to yielding $\left(P_{cr}\leq P\leq P_{y}\right);$ (III) post-yielding stage $\left(P_{y}<P<P_{u}\right)$. Three characteristic points in the load versus deflection curve include the cracking point (*∆_cr_*, *P_cr_*), yield point (*∆_y_*, *P_y_*) and peak point (*∆_u_*, *P_u_*).

**Figure 13:**
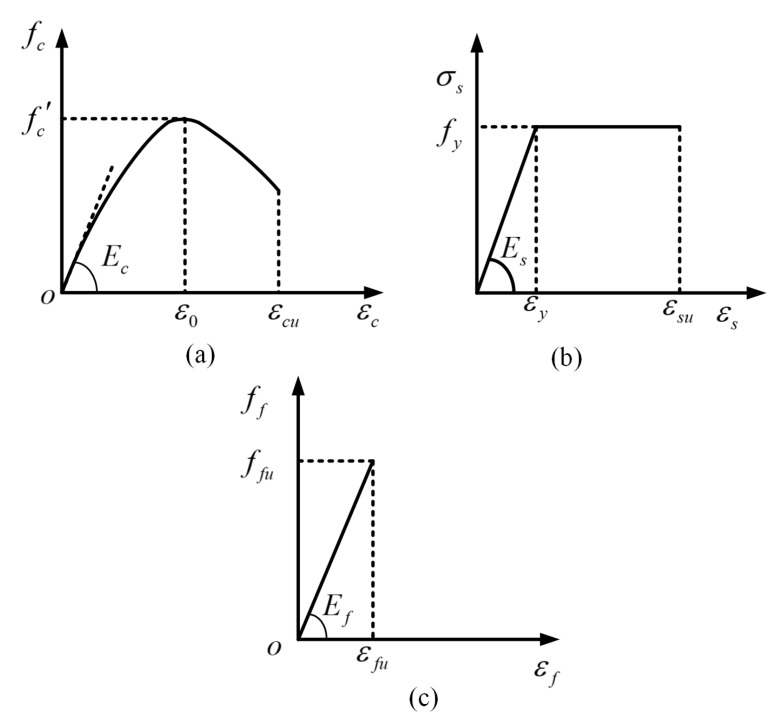
Constitutive relationships: (**a**) concrete in compression; (**b**) steel bar; and (**c**) BFRP.

Figure 13 presents the constitutive relationships of the concrete, steel bars and BFRP implemented in this study. In order to avoid shear failure, transverse steel bars were installed in the tested beams. Therefore, the responses of these beams depended also on the confinement of the concrete [34,51,52]. A widely applied constitutive relationship of confined concrete under compression was utilized [53], which can be expressed as

(3) fcεc=fc′2εcε0−2εcε02  for 0≤ε0≤εcu

where *ε_c_* and *f_c_* are the compressive strain and stress of concrete; *ε_cu_* and *ε*_0_ are the ultimate compressive strain and peak strain and are taken as 0.0033 and 0.002, respectively; and $f_{c}^{\prime }$ is the cylinder compressive strength of concrete.

The tensile stress–strain relationships for the steel bars and BFRP can be expressed by Equations (4) and (5), respectively [32,47]:

(4) $$\sigma_{s}=\left\{\begin{array}{c} E_{s}\varepsilon_{s}\;\;\;\mathrm{for}\;0\leq \varepsilon_{s}\leq \varepsilon_{y}\\ f_{y}\;\;\;\;\;\;\;\;\;\mathrm{for}\;\varepsilon_{y}\leq \varepsilon_{s}\leq \varepsilon_{su} \end{array}\right.$$

(5) $${\;\sigma }_{f}=E_{f}\varepsilon_{f\;\;}\mathrm{for}\;0\leq \varepsilon_{f}\leq \varepsilon_{fu}$$

where *ε_s_* and *σ_s_* are the tensile strain and stress of steel bar; *ε_y_* and *f_y_* are the yield strain and strength of the steel bar; *E_s_* is the elastic modulus of the steel bar; *ε_su_* is the ultimate tensile strain of the steel bar; *ε_f_* and *σ_f_* are the tensile strain and stress of BFRP; *ε_fu_* is the ultimate tensile strain of BFRP; and *E_f_* is the elasticity modulus of BFRP.

#### 4.1.1. Cracking Point

Based on conventional theories, the cracking moment (*M_cr_*) and cracking load (*P_cr_*) can be obtained by the following formulae [9,54,55,56]:

(6) $$M_{cr}=W_{0}f_{r}$$

(7) $${\;f}_{r}=0.62\sqrt{f_{c}^{\prime }}$$

(8) $${\;P}_{cr}=\frac{{2M}_{cr}}{a}$$

where *M_cr_* refers to the cracking moment, *f_r_* refers to the concrete modulus of rupture, *W_0_* represents the converted section resistance moment, *a* is the effective span of the beam, and *h* and *b* refer to the depth and width of the beam, respectively.

#### 4.1.2. Yield Point

The traditional section analysis can be adopted by assuming the steel yields before flexural failure. At the yielding stage, based on the force equilibrium of the cross-section in Equation (9) [9,54], the yielding moment *M_y_* can be calculated by Equation (10).

(9) $$k_{1}f_{c}^{\prime }bc_{y}+A_{s}^{\prime }E_{s}^{\prime }\varepsilon_{s}^{\prime }=f_{y}A_{s}+A_{f}E_{f}\varepsilon_{f}$$

(10) $$M_{y}=f_{y}A_{s}\;\;\left(d-k_{2}c_{y}\right)+A_{f}E_{f}\varepsilon_{f}\left(h-k_{2}c_{y}\right)+A_{s}^{\prime }E_{s}^{\prime }\varepsilon_{s}^{\prime }(k_{2}c_{y}-d^{\prime })$$

where *A_s_* and $A_{s}^{\prime }\;$ represent the area of tension and compression rebars. *A_f_* is the area of BFRP, $\;E_{s}^{\prime }$ and $E_{f}$ represent the elastic modulus of compression rebars and BFRP sheets, $\varepsilon_{f}$ and $\varepsilon_{s}^{\prime }$ represent the strain of BFRP sheets and compression reinforcement, *d* denotes the distance between the centroid of the tension rebars and the top concrete fiber, *d*’ refers to the distance from center of compression rebars to the top concrete fiber, *c_y_* represents the depth of the neutral axis when the tension rebars are yielding, and *k_1_* and *k_2_* are the parameters and can be calculated by Equations (11) and (12) [57,58].

(11) $$k_{1}=\frac{1}{f_{c}^{\prime }\varepsilon_{c}}\int_{0}^{\varepsilon_{c}}f_{c}\left(\varepsilon \right)d\varepsilon =\frac{\varepsilon_{c}}{\varepsilon_{0}}(1-\frac{\varepsilon_{c}}{{3\varepsilon }_{0}})$$

(12) $$k_{2}=(\frac{1}{3}-\frac{\varepsilon_{c}}{12\varepsilon_{0}})/(1-\frac{\varepsilon_{c}}{{3\varepsilon }_{0}})$$

According to the plane section assumption, the strains in the compression rebar, compression concrete and FRP sheet can be calculated by Equations (13)–(15) [58] as follows.

(13) $$\varepsilon_{s}^{\prime }=\frac{c_{y}-d_{c}}{d-c_{y}}\varepsilon_{y}$$

(14) $$\varepsilon_{c}=\frac{c_{y}}{d-c_{y}}\varepsilon_{y}\leq 0.0033$$

(15) $$\varepsilon_{f}=\frac{h-c_{y}}{d-c_{y}}\varepsilon_{y}$$

in which *ε_y_* is the yielding strain in tension reinforcement bars and $\varepsilon_{y}=f_{y}/E_{s}$.

#### 4.1.3. Peak Point

Based on the force equilibrium of the cross-section in Equation (16), the ultimate moment *M_u_* can be calculated using Equation (17).

(16) $$k_{1}f_{c}^{\prime }bc_{u}+A_{s}^{\prime }E_{s}^{\prime }\varepsilon_{s}^{\prime }=f_{y}A_{s}+A_{f}E_{f}\varepsilon_{f}$$

(17) $$M_{y}=f_{y}A_{s}\;\;\left(d-k_{2}c_{u}\right)+A_{f}E_{f}\varepsilon_{f}\left(h-k_{2}c_{u}\right)+A_{s}^{\prime }E_{s}^{\prime }\varepsilon_{s}^{\prime }(k_{2}c_{u}-d^{\prime })$$

where *k*_1_ and *k*_2_ are determined by the same equations as in stage II, *c_u_* represents the depth of the neutral axis when the load reaches ultimate load. *ε_f_* is the BFRP strain when the beam fails. *ε_f_* equals to the ultimate FRP strain *ε_fu_* when the beam fails due to FRP rupture.

(18) $$\varepsilon_{f}=\varepsilon_{fu}$$

When the failure is due to FRP debonding, the debonding strain *ε_f_* can be estimated by Equations (19) and (20) according to the ACI equation [58,59]:

(19) $$\varepsilon_{f}=k_{m}\varepsilon_{fu}\;\;$$

(20) $$k_{m}=\left\{\begin{array}{c} \frac{1}{60\varepsilon_{fu}}\left(1-\frac{nE_{f}t_{f}}{360000}\right)\leq 0.90\;\;\;\;\;\;\;nE_{f}t_{f}\leq 180000\\ \frac{1}{60\varepsilon_{fu}}\left(\frac{90000}{nE_{f}t_{f}}\right)\leq 0.90\;\;\;\;\;\;\;\;\;\;\;\;\;\;\;\;nE_{f}t_{f}>180000 \end{array}\right.$$

in which *n* is the number of BFRP layers.

Table 6 lists the predicted and measured characteristic loads as well as the ratios of predicted values to experimental results. The predicted characteristic loads showed a reasonably good correlation with the experimental results. The ratios of predicted values to experimental results of the beams strengthened using neat epoxy were slightly greater than that of the beams strengthened with MWCNT-modified epoxy. The reason should be that only the elastic modulus of BFRP sheets with and without MWCNTs could be used to reflect the difference between the two types of adhesives in the model, and the crack-bridging and pull-out effects of carbon nanotubes infiltrated into the concrete were not reflected.

### 4.2. Deflection Prediction

To calculate the deflection after the cracking point, an effective moment of inertia $I_{e}$ is used in the ACI 318-11 [56] and the ACI 318-05 [60]:

(21) $$I_{e}=\;{\left(\frac{M_{cr}}{M}\right)}^{3}I_{g}+\left[1-{\left(\frac{M_{cr}}{M}\right)}^{3}\right]I_{cr}\leq I_{g}\;\;\mathrm{for}\;\;M\geq \;M_{cr}$$

where ${\;I}_{g}$ is the gross moment of inertia and $I_{cr}$ is the cracked moment of inertia. Previous research showed that the difference between the calculated short-term deflection of FRP RC beams and the actual value is large when applying this relationship [61]. In this study, by comparing the calculated value of deflection with the experimental results, Equation (21) could be utilized to accurately calculate mid-span deflection of the FRP RC beams from the concrete cracking to steel yielding. However, there was a large gap between the predicted deflection value and the experimental results after steel yielding of the RC beams. It should be more reasonable to take a different $I_{e}$ in stage III. In this study, the following Equations (22)–(31) are recommended to calculate the appropriate $I_{e}$, in which the $I_{e}$ of stage II were taken from the ACI 318-11 [56] and the ACI 318-05 [60], and those of stage I and stage III were taken from references [62,63].

(22) $$I_{e}=\left\{\begin{array}{c} I_{unc}\;\;\;\;\;\;\;\;\;\;\;\;\;\;\;\;\;\;\;\;\;\;\;\;\;\;\;\;\;\;\;\;\;\;\;\;\;\;\;\;\;\;\;\;\;\;\;\;\mathrm{for}\;M<M_{cr}\;\;\;\\ {\left(\frac{M_{cr}}{M}\right)}^{3}I_{g}+\left[1-{\left(\frac{M_{cr}}{M}\right)}^{3}\right]I_{cr}\leq I_{g}\;\;\;\;\;\mathrm{for}\;M_{cr}\leq M\leq M_{y}\;\;\\ \frac{I_{cr,sh}}{\left[1-\left(1-\frac{I_{cr,sh}}{I_{e,y}}\right)\lambda \right]}\;\;\;\;\;\;\;\;\;\;\;\;\;\;\;\;\mathrm{for}\;M>M_{y} \end{array}\right.$$

(23) $$I_{unc}=\frac{by_{0}^{3}}{3}+\frac{b{\left(h-y_{0}\right)}^{3}}{3}+n_{s}A_{s}{\left(d-y_{0}\right)}^{2}+n_{f}A_{f}{\left(h-y_{0}\right)}^{2}$$

(24) $$I_{g}=\frac{{bh}^{3}}{12}$$

(25) $$I_{cr}=\frac{{bd}^{3}}{3}k^{3}+(n_{s}A_{s}+n_{f}A_{f})d^{2}{\left(1-k\right)}^{2}$$

(26) $$k=\sqrt{{2(\rho }_{f}n_{f}+\rho_{s}n_{s}\;)+{{(\rho }_{f}n_{f}+\rho_{s}n_{s}\;)}^{2}}{-(\rho }_{f}n_{f}+\rho_{s}n_{s}\;)$$

(27) $$\rho_{f}=\frac{A_{f}}{bh}$$

(28) $$\rho_{s}=\frac{A_{s}}{bd}$$

(29) $$n_{f}=\frac{E_{f}}{E_{c}}$$

(30) $$n_{s}=\frac{E_{s}}{E_{c}}\;\;$$

(31) $$\lambda =\frac{M_{y}}{M}\left(1-\sqrt{1-\frac{M_{y}}{M}}\;\;\right),\;\;\;\;\;M_{y}<M\leq \;M_{u}$$

(32) $$\Delta =\frac{Pa}{48E_{c}I_{e}}(3L^{2}-4a^{2}\;)$$

in which *n_f_* and *n_s_* are obtained by Equations (29) and (30), respectively; *y*_0_ is the depth of the neutral axis. In stage II, *M_cr_*,${\;I}_{g}$ and $I_{cr}$ are calculated by Equations (6) and (24)–(30). In stage III, λ is calculated by Equation (31). ${\;I}_{e,y}$ is obtained by substituting the yield bending moment *M_y_* for *M* in Equation (22). The calculation of $I_{g}$ in stage III takes into account the influence of strain hardening of steel bars.${\;I}_{cr,sh}$ is calculated using Equation (25), in which *E_s_* equals *E_s,sh_*. *E_s,sh_* may be taken as *E_s_*/10 if no other information is available [62]. The mid-deflection can be simply calculated using Equation (32) after ${\;I}_{e}$ has been obtained.

Table 7 lists the predicted and measured characteristic deflections as well as the ratios of predicted values to experiment results. The predicted characteristic deflections indicated a reasonably good correlation with the experimental results.

## 5. Conclusions

The flexural behavior of RC beams strengthened with BFRP sheets using MWCNT-modified epoxy was studied by four-point bending beam tests, including failure mode, load–deflection relationships, development process of crack width, energy absorption capacity, stiffness and ductility, as well as the strain response of concrete, rebar and BFRP sheets. The enhancement mechanisms of MWCNTS on the flexural behavior were analyzed by detailed SEM observations. Some main conclusions were as follows:

1. The reinforced specimens failed due to intermediate IC debonding after steel yielding, and the failure occurred within the concrete.
2. The BFRP sheets bonded using the MWCNT-modified epoxy more effectively constrained crack development of strengthened beams, as well as delayed the IC debonding and the eventual rupture of BFRP sheets.
3. The yielding load and, ultimate load, as well as the ultimate deflection of the reinforced specimens were enhanced by the MWCNT-modified epoxy. When the beam was reinforced with two-layer BFRP sheets, the yielding load and ultimate load, as well as the ultimate deflection of the strengthened specimen using MWCNT-modified epoxy increased by 7.4%, 8.3% and 18.2%, respectively, compared with the beam strengthened using pure epoxy.
4. A significant enhancement of the flexural stiffness, energy absorption capacity and ductility of the reinforced specimens using BFRP sheets was achieved by using MWCNT-modified epoxy. When the beam was reinforced with two-layer BFRP sheets, the beam strengthened using MWCNT-modified epoxy significantly enhanced the post-yielding flexural stiffness, energy absorption capacity before the ultimate load, and deflection ductility index by 22.6%, 29.1% and 14.3%, respectively, compared with the beam strengthened using pure epoxy.
5. The SEM images indicated the MWCNTs could penetrate into the concrete pores. Both crack-bridging and pull-out of high-strength MWCNTs consumed more energy, which greatly increased the interfacial fracture energy and remarkably enhanced flexural behavior of the strengthened beams.
6. Based on the ACI Code, an analytical model was proposed for calculating the flexural capacity and mid-deflection of RC beams strengthened with FRP sheets using MWCNT-modified epoxy. The predicted characteristic loads and characteristic deflections showed a reasonably good correlation with the experimental results. The predicted values of the beams strengthened using MWCNT-modified epoxy were slightly more reliable and conservative than those of the beams strengthened with neat epoxy. The analytical model can provide theoretical references for practical application.

## Figures and Tables

**Table 1:** Material properties of longitudinal steel bars.

Type	Diameter (mm)	Yield Strength (MPa)	Ultimate Strength (MPa)	Yield Microstrain (με)	Modulus of Elasticity (GPa)
HPB300	8	300	420	1430	210
HRB335	12	400	550	2100	200

**Table 2:** Specimen details.

Scheme	Number of BFRP Layers	Adhesive
CB	0	
B-Neat-1	1	Neat epoxy resin
B-CNT-1	1	MWCNT-modified epoxy resin
B-Neat-2	2	Neat epoxy resin
B-CNT-2	2	MWCNT-modified epoxy resin

**Table 3:** Summary of test results.

Specimen Number	Cracking Load *P_cr_* (KN)	Yield Load *P_y_* (KN)	Ultimate Load *P_u_* (KN)	Deflection at Cracking Load ${∆}_{cr}$ (mm)	Deflection at Yield Load ${∆}_{y}$ (mm)	Deflection at Ultimate Load ${∆}_{u}$ (mm)	*E_I_* (KN/mm)	*E_II_* (KN/mm)
CB	12.87	45.95	64.65	0.52	3.95	29.52	9.17	0.94
B-Neat-1	12.96	52.98	72.62	0.44	4.09	14.44	11.11	1.08
B-CNT-1	13.04	57.01	75.02	0.51	4.11	16.41	11.36	1.26
B-Neat-2	13.11	60.59	84.35	0.61	4.08	17.24	13.33	1.33
B-CNT-2	13.15	65.06	91.38	0.63	4.23	20.38	13.64	1.63

Notes: *E_I_* and *E_II_* denote the flexural stiffness before and after yielding.

**Table 4:** Energy absorption capacity.

Specimen Number	J_c_ (kN·mm)	*J_y_* (kN·mm)	*J_u_* (kN·mm)
CB	2.75	103.19	1578.33
B-Neat-1	3.33	147.72	809.29
B-CNT-1	3.40	150.73	970.43
B-Neat-2	3.41	135.12	1116.05
B-CNT-2	3.51	158.62	1440.91

Notes: J_c_, *J_y_* and *J_u_* represent the energy absorption before the cracking, yield and ultimate load, respectively.

**Table 5:** Ductility index.

Specimen Number	$\mu_{\Delta }$	$\mathrm{Ratio}\;\mu_{\Delta }$$/(\mu_{\Delta })$_CB_ to CB Specimen	$\mu_{E}$	$\mathrm{Ratio}\;\mu_{E}$$/(\mu_{E})$_CB_ to CB Specimen
CB	7.47	1	12.72	1
B-Neat-1	3.53	0.47	5.48	0.36
B-CNT-1	3.99	0.53	6.44	0.42
B-Neat-2	4.23	0.56	7.20	0.54
B-CNT-2	4.82	0.64	8.22	0.59

**Table 6:** Calculated and experimental capacity of characteristic points.

Specimen Number	*P_cr,the_* (KN)	*P_cr,exp_* (KN)	*P_cr,the_/* *P_cr,exp_*	*P_y,the_* (KN)	*P_y,exp_* (KN)	*P_y,the_/* *P_yexp_*	*P_u,the_* (KN)	*P_u,exp_* (KN)	*P_u,the_/* *P_u,exp_*
CB	12.23	12.87	0.95	53.44	47.95	1.11	56.68	64.65	0.92
B-Neat-1	12.23	12.96	0.94	55.94	52.98	1.06	64.33	72.62	0.93
B-CNT-1	12.25	13.04	0.94	56.15	57.01	0.98	65.19	75.02	0.91
B-Neat-2	12.26	13.11	0.94	57.61	60.59	0.95	74.31	84.35	0.88
B-CNT-2	12.26	13.15	0.93	57.98	65.06	0.89	75.72	91.38	0.83

**Table 7:** Calculated and experimental deflection of characteristic points.

Specimen Number	*Δ_cr,the_* (mm)	*Δ_cr,exp_* (mm)	*Δ_cr,the_/* *Δ_cr,exp_*	*Δ_y,the_* (mm)	*Δ_y,exp_* (mm)	*Δ_y,the_/* *Δ_y,exp_*	*Δ_u,the_* (mm)	*Δ_u,exp_* (mm)	*Δ_u,the_/* *Δ_u,exp_*
CB	0.43	0.52	0.83	3.49	3.95	0.88	29.85	29.52	1.01
B-Neat-1	0.41	0.48	0.85	3.60	4.09	0.88	16.03	14.44	1.11
B-CNT-1	0.41	0.48	0.85	3.61	4.11	0.88	16.26	16.41	0.99
B-Neat-2	0.41	0.47	0.87	3.67	4.08	0.90	17.39	17.24	1.01
B-CNT-2	0.41	0.46	0.89	3.68	4.21	0.87	17.60	18.77	0.94

## Data Availability

The raw/processed data required to reproduce these findings cannot be shared at this time as the data also form part of an ongoing study.
